# Vulnerability to Sexually Transmitted Infections (STI) / Human Immunodeficiency Virus (HIV) among adolescent girls and young women in India: A rapid review

**DOI:** 10.1371/journal.pone.0298038

**Published:** 2024-02-14

**Authors:** Sohini Paul, Anupam Sharma, Radhika Dayal, Mahika Mehta, Sudeshna Maitra, Kuhika Seth, Monal Nagrath, Sowmya Ramesh, Niranjan Saggurti

**Affiliations:** 1 Population Council Consulting Pvt Ltd, Delhi, India; 2 Indian Institute of Technology, Gandhinagar, Gujarat, India; 3 International AIDS Vaccine Initiative, Delhi, India; 4 Population Council Institute, Delhi, India; 5 Population Council, Delhi, India; Noida International University, INDIA

## Abstract

Limited evidence is available on the vulnerability of Adolescent Girls and Young Women (AGYW) to sexual risk behaviour and STI/HIV. Though there are almost no statistics available on vulnerability, related literature suggests that AGYW have low awareness about sexual risk behaviour/ transmission and the prevalence of STI/HIV, making them vulnerable. We conducted a rapid review of peer-reviewed studies addressing transmission network, prevalence, incidence awareness, common determinants of sexual risk behaviour/STI/HIV, health-seeking behaviour and existing interventions addressing the situation among AGYW (age 15–24) to inform the evidence gap in this crucial area of research. We registered the study in PROSPERO (CRD42023403713). We developed detailed inclusion/exclusion criteria, searched JSTOR, PubMed, Google Scholar, Science Direct and Population Council Knowledge Commons databases and followed the guidance from Cochrane Rapid Review to develop the rapid review. We also searched the bibliography of the included studies. We included the English language peer-reviewed quantitative, qualitative, mixed method studies published from Jan 1 2000 to Mar 31 2023. Six reviewers extracted data, and the seventh reviewer independently assessed the quality. Ninety-six studies met the inclusion criteria. We used descriptive statistics and narrative synthesis methods for data analysis. We also conducted a Risk of Bias Assessment (RoB) to check the quality of the included studies. Inadequate literature was found on the transmission network. Prevalence and awareness of STI/HIV are low among AGYW. However, Female Sex Workers, sex-trafficked women or drug users in this age group suffer more. Age, education, income, relationship dynamics with spouses/partners, multiple partners, and substance use are crucial in determining STI/HIV. Traditional sources of health seeking are more popular than formal sources because of social stigma. Mass media campaigns, community mobilization programs, and life skills training programs increase awareness about HIV, condom use and self-efficacy. The inclusion of only English language studies and not conducting meta-analysis because of high heterogeneity are some of the limitations of the study.

## Introduction

Sexually Transmitted Infections (STI) / Human Immunodeficiency Virus (HIV)vulnerability among adolescent girls and young women (AGYW) is a serious concern in developing nations, even though HIV prevalence has declined globally by 38% during 2010–2022 [1]. India has approximately 2.3 (1.8–3.0) million people living with HIV/AIDS; most of these are individuals over the age of 15 years [2], and 6% of the adult population suffers from one or more STIs at any one time [3]. While 39 million people have HIV globally [WHO 2023a], out of which 1.75 million are adolescents [4]. However, no statistics are available for the AGYW community on their vulnerability to STI and HIV infection, a major gap in the backdrop of Sustainable Development Goal 3.3, which aims to end the HIV epidemic by 2030 [5]. Adolescent Girls and Young Women (AGYW) are prone to indulging in risky sexual behaviours in the transitional phase of life towards adulthood, which makes them susceptible to sexual coercion, violence, and increased vulnerability to STI/HIV/AIDS [6].

In India, almost 23% of women were reportedly married below the legal age. About 23.7% of girls aged 15–19 have had a live birth, the median number of days of the last sexual encounter reported by girls (15–19 years) was 7.7 days, and only about 33.7% of women in the age group 20–24 years (who had two or more partners in the last 12 months) had reported using a condom during sexual encounters, and about 7.7% of ever-married girls aged 18–24 years have reported experiencing sexual violence [7]. Comprehensive knowledge of HIV/AIDS and STIs among AGYW is also limited among adolescents, making them vulnerable to risky sexual behaviour. National Family and Health Survey (NFHS) reports that 22% of young women and 32% of young men aged 15–24 years have comprehensive knowledge of HIV [7]. Furthermore, the risk of HIV among adolescents could be compounded by their incomplete and inaccurate knowledge [8]. A longitudinal study conducted in Uttar Pradesh reveals limited HIV knowledge among unmarried and married older girls (30–37%) as compared to older boys (57%) aged 15–19 years [9].

Global evidence shows AGYW are disproportionately vulnerable to HIV infection due to greater physiological risk, gender inequality, unequal gender norms and gender-based violence, including intimate partner violence and early marriages [10]. However, there is a lack of comprehensive evidence in the Indian context about the vulnerability of the AGYW community towards HIV/ STI infection.

Most relevant literature in India has focused on high-risk and key populations such as female sex workers, men who have sex with men and injecting drug users or trafficking victims [11–13]. There has been limited research on adolescent and young populations/non-key populations and their vulnerability to HIV risks. To the best of our knowledge, no other literature reviews are available on this topic except a study protocol published by Patsani et al. [14].

Against such a backdrop, we aimed to conduct a rapid review of the literature on the vulnerability of adolescent girls and young women to STI/ HIV infection. We conducted a rapid review for the following broad domains: (i) Spatial distribution or concentration of HIV/ STI infection, (ii) awareness, prevalence, and incidence of sexually transmitted infections / HIV, (iii) common determinants of STI, HIV infection and sexual risk behaviours, and (iv) health-seeking behaviour and interventions (and their effectiveness) to prevent/communicate about STIs / HIV and sexual risk behaviours among AGYW in India.

## Methods

We designed the rapid review with guidance from the Cochrane Rapid Review [15] and registered the study with the International Prospective Register of Systematic Reviews (PROSPERO), registration ID: CRD42023403713 (Refer to S1 Appendix). We attached the detailed study protocol as a supplementary document (Refer to S1 File), which we followed while developing the review article. Developing the planned search strategy and selecting search terms and electronic databases was guided by other rapid and systematic reviews and publications on HIV infections among AGYWs [16–23]. We used systematic search strategies in the rapid review, but it is limited to particular aspects to provide a time-sensitive assessment of the available literature [24].

### Literature search strategies and study selection

We searched JSTOR, PubMed, Google Scholar, Science Direct and Population Council Knowledge Commons databases for English language articles, reports, reviews, and research briefs published between January 2000 and March 2023, focusing on adolescent girls and young women (AGYW) in the age group of 15–24. We developed detailed inclusion/ exclusion criteria mentioned in Table 1. We included evaluation studies that used Randomized Control Trials (RCTs), quasi‐experimental design (fixed-effects, regression discontinuity, instrumental variables analysis and difference-in-difference analysis), studies that used longitudinal data sets, qualitative studies, and literature review articles. Grey literature, research briefs and peer-reviewed published articles are considered for the review. We excluded the studies that are not focused on India or women, not within the 15–24 age group. We searched articles for key and non-key populations; the key population included AGYW involved with the sex trade or in a same-sex relationship. The non-key population has AGYW, who are not considered the key population.

**Table 1:** Inclusion and exclusion criteria.

Criterion	Include	Exclude
Language	Published in English	Not in English
Population	Adolescent girls and young women	Men
	15–24 age group	Girls below the age of 15
		Women more than age 25
Spatial distribution	India—national-level studies and state-specific studies	Other than India
Knowledge and incidence of STI/ RTI/HIV	Knowledge of STI/RTI/HIV	Awareness/prevalence/incidence of any disease other than STI/RTI/HIV
	Prevalence of STI/RTI/HIV	
	Incidence of STI/RTI/HIV	
	Sources of information for STI/RTI/HIV	
Determinants of STI/ HIV	Focusing on determinants of STI/HIV/ sexual risk behaviour at the individual level, community or institution level, societal level	The direction of association between determinants and outcomes not clear or inconclusive
Health seeking behaviour	Formal service providers	Does not consider any of the providers
	Traditional healthcare providers	
	Informal sources	
	Prevention programs	
	Participatory training programs	
Interventions	Evaluation of single or multiple component interventions compared against appropriate counterfactual	Insufficient details on intervention
	Outcomes will include the following -	Evaluation not adequately designed.
	HIV knowledge and prevalence	
	Risky sexual behaviour	
	Forced sex	
	Self-efficacy/self-negotiation/ Positive gender attitude	
Timing	Studies conducted between 1st January 2000 to 31st March 2023	Studies conducted before 2000 or after March 2023
Study design	Empirical studies from	Editorials
	Randomised Control Trial	Commentaries
	Quasi experimental design	Research Brief
	Used cross-section or longitudinal data	
	Methods used—qualitative/ quantitative/mixed	
	Systematic reviews	
	Published report	
	Published journal article	
	Grey literature	

We finalized the search keywords using a combination of search terms like Adolescent Girls and Young Women, HIV, Sexual and Reproductive Health, Sexual and Reproductive Health Knowledge, Sexual and Reproductive Health Awareness, Sexual and Reproductive Health Risks, Behavioural Interventions, and others. The final search strings are attached as support material (Refer to S1 Table).

### Data extraction, synthesis, analysis, and quality assessment

Starting from searching keywords to synthesizing the extracted content, we adhered to the guidelines of the Cochrane group [15]. Six researchers (RD, AS, SM, MM, KS, MN) manually and independently surveyed the titles, abstracts, keywords, and full texts of the articles, performed relevancy checks and finalized the list of published articles and grey literature. Minimal data set is included as supporting information (Refer to S2 Table). They also extracted the data, and two team members (AS and SM) checked for duplicate articles. One team member (SP) independently performed the quality assessment of 50% of the selected papers manually. For all the articles, data is extracted on the year of publication, type of publication (peer-reviewed journal article, report or research brief), target geography, objective or research questions addressed in the article, duration of data collection (if quantitative), study design–quasi-experimental, Randomised Control Trial, Cross-Sectional approaches for quantitative studies, narrative synthesis, descriptive through case studies or In-Depth Interviews (IDIs) or Focus Group Discussions (FGD) for qualitative studies. Additional data included sample size and sampling strategy (random/ stratified/ purposive, convenience), age range and socio-demographic profile of the respondents. Then, we extracted data based on four research questions: spatial distribution or transmission network of STI / HIV /sexual risk behaviour, awareness, prevalence and incidence of STI/ HIV along with outcome measure and effect sizes, prevalence and incidence of sexual risky behaviour, determinants of STI/ HIV, strategies/sources to influence/encourage health-seeking behaviour, intervention strategies to prevent sexual risk behaviour/ STI and HIV, intervention/exposure measures, including frequency of intervention, intervention components, effects of intervention (with effect sizes). Following the Cochrane guidelines [15], designated reviewers extracted data using a piloted form. Further, other reviewers checked for the correctness and completeness of the extracted data. Data extraction was limited to a set of required data items based on our a priori research questions.

Given the heterogeneity of the study design, we did not conduct any pooled analysis. Instead, we used the narrative synthesis method [25, 26] guided by the review questions. Four coders (AS, RD, SM, MM) coded the text line-by-line in the first step. Then, they derived the descriptive themes following four research questions of HIV/ STI vulnerability of the AGYW community–spatial distribution of HIV/ STI infection, awareness, prevalence and incidence of HIV/ STI infection, determinants of sexual risk behaviour, HIV/ STI infections, health-seeking behaviour or intervention strategies to prevent or communicate about STI/ HIV or sexual risk behaviour and analyzed the review findings accordingly. We also used descriptive statistics, including frequency tables and graphs, to tabulate the distribution of articles across different outcomes. Approximately one-third of the extracted studies focused on high-risk populations such as men having sex with men (MSM) and transgender and commercial sex workers and assessed their sexual risk behaviour and risk and prevalence of STI/HIV along with health-seeking and intervention strategies. We included the analysis of high-risk populations under each of the four descriptive themes.

We developed Risk of Bias (ROB) assessment tools for this rapid review applicable to qualitative and quantitative studies. Following the Cochrane guidelines for data quality, we conducted a rigorous risk of bias assessment, where we used valid risk of bias tools (separately for qualitative and quantitative studies). The ROB assessment tool for quantitative studies was adapted from the Effective Public Health Practice Project (EPHPP) Quality assessment tool [27]. The ROB assessment tool for qualitative studies was adopted from Consolidated Criteria for Reporting Qualitative Research (COREQ) guidelines [28]. We assessed each article and summarised the findings attached as support materials indicating the overall quality of the articles included in this rapid review. For the ROB assessment of the mixed methods studies, we used the quantitative and qualitative tools for the quantitative and qualitative sections, respectively. Five reviewers (SP, AS, RD, SM and MM) conducted the ROB assessment. The quality of the risk of bias assessment exercise is ensured by another team member, SR, independently by randomly checking approximately 15% of the total studies. Discrepancies were resolved to attain high inter-rater reliability. We also have limited the risk of bias ratings to the most important outcomes, with a focus on those most important for decision-making. The authors were in continuous discussions with experts from the funding agency, and the reviews received at each stage were incorporated adequately throughout the process of the rapid review.

## Results

Of 242 records screened, 96 articles met our inclusion criteria. These studies were conducted either across India or focused on specific states, including Maharashtra, Rajasthan, Jharkhand, Haryana, Karnataka, Gujarat, Punjab, Andhra Pradesh, Bihar, Tamil Nadu, Himachal Pradesh, Mizoram, Uttar Pradesh, Kerala, and Delhi. They included quantitative (n = 68), qualitative (n = 20) and mixed method (n = 6) study designs. Furthermore, we had two articles that focused on reviews of conceptual or implementation frameworks. Of the included studies, 3 reported spatial distribution of HIV/ STI infection, 23 on awareness/ prevalence/ incidence of risky sexual behaviour or HIV/ STI, and 22 mentioned the determinants of HIV/ STI/ sexual risk behaviour. At the same time, 27 dealt with help-seeking behaviour or intervention strategies to prevent HIV/ STI. While the rest 21 studies overlap across different research questions. We reported the distribution of studies across four research questions in Table 2.

**Table 2:** Distribution of studies across four research questions.

Research question	Number of articles
RQ1	3
RQ2	23
RQ3	22
RQ4	27
RQ1, RQ2	5
RQ1, RQ3	1
RQ2, RQ3	10
RQ2, RQ4	2
RQ3, RQ4	2
RQ1, RQ2, RQ3	1
**Grand Total**	96

**Fig 1:**
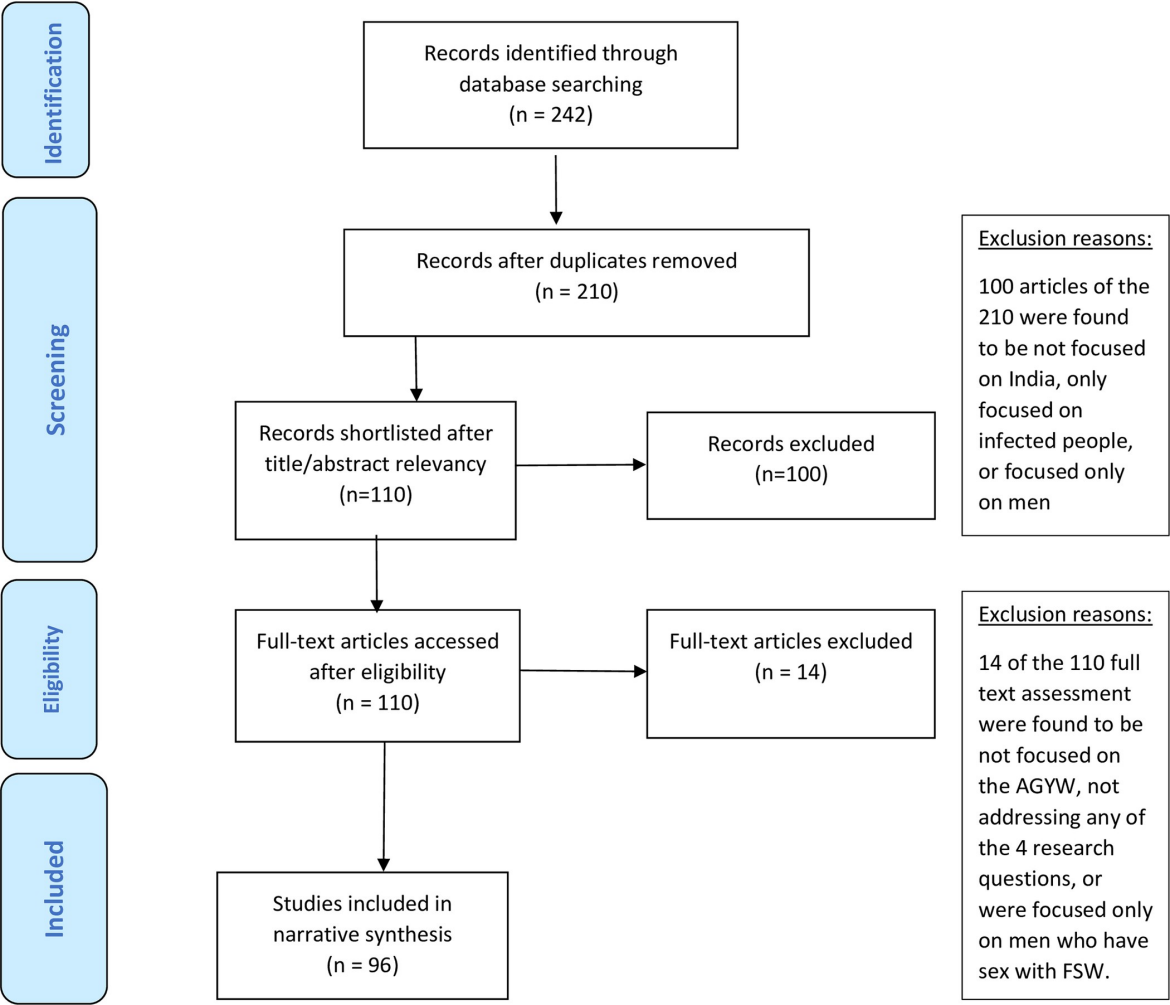
PRISMA flow diagram for journal articles.

Following the PRISMA flow diagram, we reported the distribution of included articles, reports, and grey literature in Fig 1. We also added PRISMA checklist for abstract (Refer to S1 Checklist) and PRISMA checklist for main manuscript (Refer to S2 Checklist) as the supporting information.

### Spatial distribution of risky sexual behaviour / HIV/ STI vulnerability and prevalence

Nine studies identified districts/states or regions with a high prevalence of HIV infection or premarital sex among the AGYW community. Following the literature, we reported the transmission network of high premarital sex and HIV prevalence among key and non-key populations in Table 3.

**Table 3:** Spatial distribution of HIV prevalence and premarital sex.

Focus	Region
High premarital sex	Central India; North India; Manipur; Nagaland; Gujarat; Uttar Pradesh; Jharkhand
High HIV prevalence	Among general population: South India; Northeast India; Southeastern coastal zone of Andhra Pradesh, Odisha, Karnataka
	Among FSWs: The western part of central India, including Gujarat, and parts of Rajasthan, Madhya Pradesh, and Maharashtra are high-risk regions.
Risk of HIV transmission among injecting drug users (IDU)	Mizoram
A higher number of trafficked girls	Karnataka; Maharashtra

Ten percent of districts, mainly in south and northeast India, observed a consistently high prevalence of HIV [29]. Prevalence of premarital sex is relatively higher in the central region of India (24% male and 5% female), followed by the northern part (19% male and 4% female). On the other hand, the prevalence of premarital sex among unmarried men and women was the lowest in the southern region. Prevalence of premarital sex is higher in Manipur (5%) and Nagaland (12%), Gujarat (3.5%), Uttar Pradesh (4.3%) and Jharkhand (3.4%) [30].

One study mentioned that the southeastern coastal zone of Andhra Pradesh is one of the vulnerable areas for HIV infection, where women are involved with the sex trade and injecting drug use [31]. Indian women and girls trafficked from the states of Karnataka and Maharashtra were more likely to test positive for HIV than those trafficked from other Indian states (P = 0.003).

One article [32] found that Mizoram in northeastern India observed the highest risk of HIV transmission among injecting drug users (IDU) (19.8% among IDU), followed by Manipur. Meghalaya has the lowest vulnerability [32].

### Awareness/ prevalence/ incidence of sexual risk behaviour/ HIV/ STI

Thirty-seven articles discuss awareness, prevalence and incidence of HIV/ STI/ sexual risk behaviour, prevalence, and incidence. Ten out of 37 articles focused on adolescent girls and young women, while the rest considered women in the reproductive age group (15–49) as the study sample. These studies were conducted in Delhi [33, 34], Maharashtra, Gujarat [35], Uttar Pradesh [36], Bihar [37] Punjab [38], Tamil Nadu [39] using primary data. At the same time, Tripathy [40] used DLHS data at the all-India level for this age group to analyze the awareness about sexual and reproductive needs. We presented the distribution of articles focusing on research question 2 in Table 4.

**Table 4:** Distribution of articles–awareness/ prevalence/ incidence of sexual risk behaviour/ STI/HIV.

Study Focus		No. of articles
Awareness	Knowledge about HIV/STI (ever heard of STI/HIV)	16
	Knowledge about STI/HIV transmission pathways	20
	Sources of information	11
	Contraception	10
Prevalence	STI	9
	HIV	13
Incidence	STI	1
	HIV	1

Most of the articles mentioned that the AGYW community had limited knowledge about the transmission of HIV or its cure. Knowledge about STIs other than HIV (Genital Herpes, Gonorrhoea or Syphilis) is even more limited. A few studies mentioned that a proportion of respondent girls are aware of transmission pathways of HIV through sexual practices with multiple partners, unsafe sex with partners and sex workers, transmission through blood transfusion or sharing of needles/syringes, and transmission from infected mother to child [33–35, 38–41]. One study stated that economic status, education, locality of residence (urban/rural), and caste are significant determinants of SRH knowledge [37]. As pointed out by [39], Of the respondents, 75% were aware of HIV/AIDS, but 39% and 19% only knew the mode of transmission and prevention of HIV infection, respectively.

Very few studies provided statistics on dating or premarital sex among AGYW. A small proportion of unmarried adolescent girls in a study from Delhi reported that they are dating (3% of 313) or are sexually active (4% of 313) [42]. Jain et al. [43] found that the mean sexual debut age is 17, and consensual sexual activity started early. Partners of the girl respondents took more initiative to form emotional (64%) and physical relationships (78%).

Eleven studies considered the prevalence or incidence of STI/HIV among the AGYW community as outcome measures [39, 44, 45]. One of these three studies [39] identified the prevalence of any symptom suggestive of Reproductive Tract Infection (RTI) or Urinary Tract Infection (UTI) among 52% of the respondents. Chandrashekarappa et al. [45] found that 7% of the respondents (pregnant mothers) were diagnosed with herpes simplex virus type 2 antibodies, 0.40% and 0,12% suffered from hepatitis B and syphilis. Fifty percent of children of HIV-infected mothers were found to be HIV-negative. One study from Meghalaya pointed out that 64% of 250 young female respondents suffered from STI symptoms like lower abdominal pain, itching in the genital area and burning micturition [44].

The average age of HIV infection is 16 years among sex trafficking victims [46]. In Karnataka, migrated female sex workers are more likely to practice safe sex under the protection of institutional set-up and less vulnerable to HIV compared to mobile or street-based FSWs based in brothels [47]. One study focusing on injecting drug users [48] from northeast India showed that HIV seropositivity is high among the respondents who share their drug-injecting syringes. Over three-quarters (n = 48) of sex-trafficked women and girls were tested for HIV; Sex trafficked women and girls lack autonomy and are rendered vulnerable to HIV infection through several means [49].

Three out of 37 studies discussed the sources of information about HIV/ STI transmission or cure, which may be informal or formal sources. Informal sources include peers and friends, while formal sources include schools [33], television, newspapers, and posters at the local health clinic [34, 41].

### Determinants of risky sexual behaviour/ HIV/ STI

Thirty-six studies reported common determinants of STI / HIV infections and sexual risk behaviours for AGYW. Some of the studies considered the age range of the respondents as 12–30 [38, 39, 42–44, 49–54]. The rest of the studies included the AGYW community as a part of the study participants comprising women of reproductive age (15–49).

Out of 36 studies, 21 are quantitative, 10 are qualitative, 4 discuss the descriptions of health profiles of AGYW, risky behaviours, and diverse characteristics of FSWs, and 1 is a review article. Quantitative techniques are applied to the cross-sectional survey data; descriptive analysis, multiple regression analysis or path analysis are generally used for analytical purposes.

A review article discussed that the determinants of vulnerability to HIV include individual factors, school, family and societal factors, and emotional and social influencers [54].

Significant determinants of sexual resilience that are the time taken before sexual debut include attitude towards premarital sex (0.638), self-esteem (0.528), and partner pressure (-0.482). Girls usually experience more parental monitoring (0.315) and are more likely to have higher self-efficacy to refuse sex (0.333). A significant predictor of vaginal intercourse for males is peer norms regarding sex (0.154) along with preparatory behaviour (0.324) [53]. Qualitative studies also mentioned that young women have poor sexual negotiation power with their partners, which may lead to unsafe sexual activities and exposure to HIV/ STI [50]. AGYW community often cannot resist unsafe sex or end an oppressive relationship due to a lack of resilience. They accept unsafe sex to retain partners and suffer from consequences. However, the correlation of sexual resilience with age, family income, education and knowledge is insignificant. One study mentioned that lack of sexual resilience in highly vulnerable areas is one of the critical determinants of unwanted pregnancies, sexually transmitted diseases, HIV/ AIDS or cervical cancer [43].

There is a significant association between the nuclear status of a family and preference for premarital sex [38]. Education and economic status are significant determinants of pre and extramarital sex of married men. Interestingly, the husband’s having had premarital sex was directly and positively associated with having had extramarital sex in the past year (0.64, p < .001) [44]. Singh et al. [30] found that the use of condoms is high at first premarital sex among educated men and women in urban areas using the fourth round of National Family Health Survey data.

Awareness of HIV is one of the significant factors (adjusted OR 4.1, 95% CI 1.03–16.3) in using condoms in marital sex [44]. Women’s autonomy, knowledge, and awareness about HIV, along with age, education, and economic status, are significant determinants of condom use or safe sex practices in Kerala and Uttar Pradesh [55]. Age and education also play essential roles in HIV transmission [56, 57]. Uneducated women had 2.3 times more risk of HIV infection compared with women with some education (95% CI 1.5–3.6; P < 0.001), as mentioned in a study focusing on the private health care sector in Puna [57].

A study among discordant couples registered in an ART centre in Karnataka found that 38% of the total sample (n = 925) did not use condoms, and transmission of infection was mainly through blood [58]. Sex workers face a high risk of HIV since male clients are not willing to use protection [49] and have low negotiation power due to fear of violence [59]. Economic insecurity and debt in this community also lead to forced and unprotected sex, which becomes an important determinant of HIV risk [51].

### Health-seeking behaviour and interventions to prevent STI/HIV

Fifteen studies addressed health-seeking behaviour. Age, caste, level of education, wealth status and exposure to marital violence are important determinants of health-seeking behaviour. Among respondents who had experienced symptoms of RTIs, 57% of married and 66% of unmarried women had not sought treatment. Two-fifths of married women had sought treatment from formal medical providers (11% from the public sector and 28% from the private sector), and one-third of unmarried women (10% and 20%, respectively). Only 3–4% of either group had relied on traditional healthcare providers or home remedies [60].

**Fig 2:**
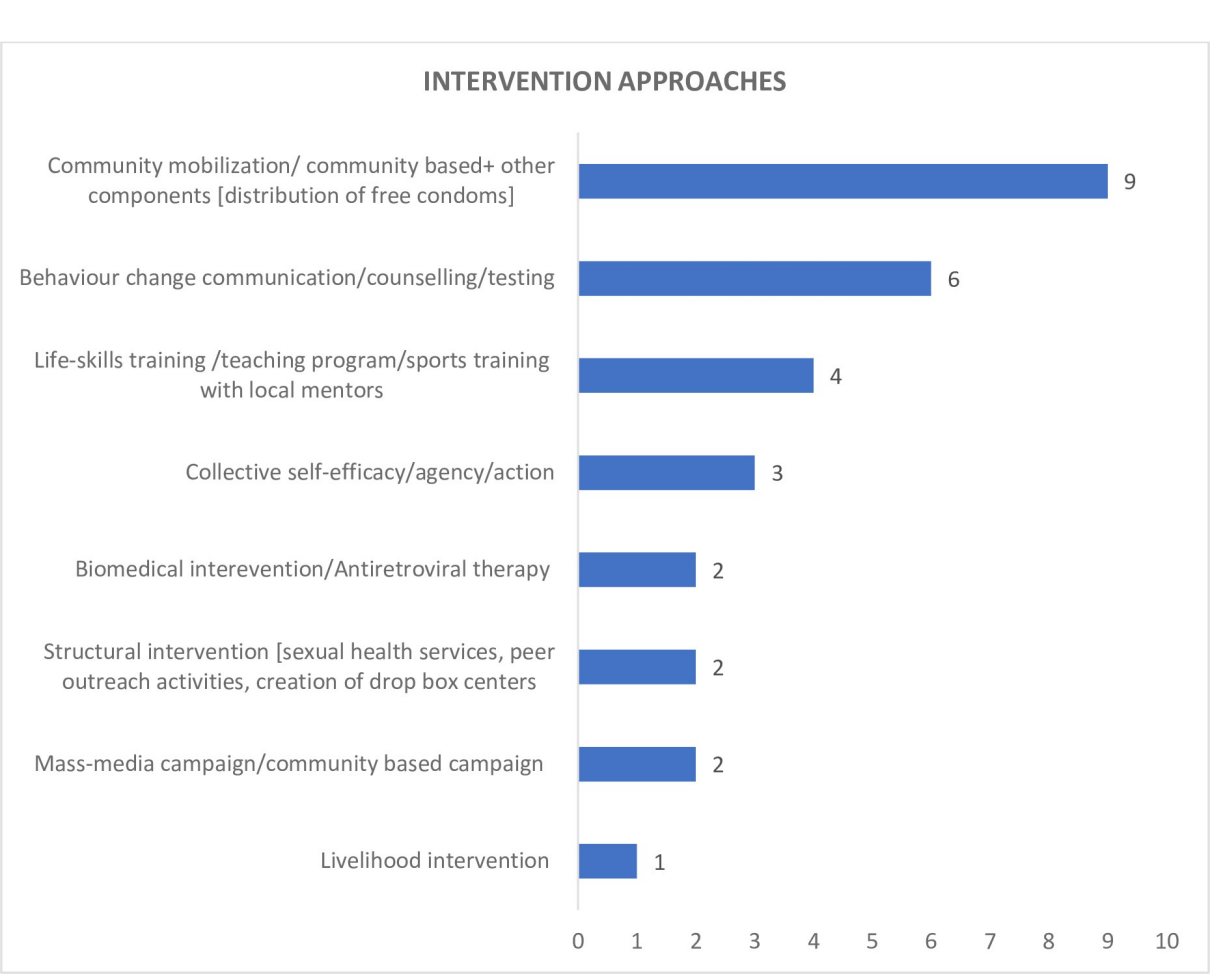
Distribution of studies across different intervention approaches.

Twenty-five studies discussed intervention on HIV/ STI awareness and analyzed whether sexual practises or HIV/ STI prevalence changed with improved awareness post-intervention. Of 25 intervention studies, eight focused on intervention for female sex workers, two targeted injectable drug users and alcoholics and 1 targeted pregnant women. One study discussed the providers’ perspective on adolescent-friendly health services [61]. We found one study which examined the correlates of health-seeking behaviour [61]. About ten were single intervention studies, while the remaining 15 were multiple component intervention studies. Eleven studies used qualitative data to mention the intervention approaches in protocols and frameworks. The distribution of studies across different intervention approaches is presented in Fig 2.

Most youth and unmarried women respondents from the District Level Household Survey expressed the importance of family life education to improve knowledge of Sexual risk behaviour, HIV and STI. The primary sources of such education include teachers, educational institutes, family members, including elder sisters or sisters-in-law, friends, and peers [62]. The community-based campaign is one of the significant determinants of HIV testing facility awareness [63]. Mass media campaigns increased HIV knowledge (22.8%) and decreased perceived risk [64]. School-based intervention programs in Himachal Pradesh reduced risk-taking behaviour among adolescent school children [65]. The intervention improved knowledge about STI transmission (85% of cases) and HIV prevention (77.5%) among non-infected women in Mumbai [66]. HIV counselling sessions helped decrease HIV prevalence among pregnant mothers attending an antenatal care clinic in Puna [57]. We reported the association between intervention strategies and primary outcomes, including risk-taking behaviour, HIV prevalence and knowledge, self-efficacy, condom use, etc., in Table 5.

**Table 5:** Intervention strategies–What worked?

Intervention strategies [n = number of articles]	Primary outcomes [positive or negative effects]
Mass-media campaigns +Teaching Program+ Behaviour change communication/counseling+ training on communication/ relationship skills [n = 4]	HIV knowledge [+]
Distribution of free condoms+ community mobilization+ structural interventions+ counseling+training on communication/ relationship skills + mass-media campaigns [n = 8]	Condom use [+]
Collective self-efficacy + Life-skills training [n = 3]	Self-efficacy [+]
Community based model+ medical and referral services +community based HIV campaign +HIV testing program [2]	HIV counselling and testing services [+]
Community mobilization+ life-skills training+ structural intervention [n = 4]	HIV prevalence [–]
Life-skills training [n = 1]	Risk-taking behaviour [–]
Mass-media campaign+ training on communication + relationship skills [n = 2]	Positive gender attitudes [+]
Mass-media campaigns+ training on communication + relationship skills+ life skills training [n = 3]	Inter-personal communication [+]
Community mobilization [n = 1]	HIV-positive cases [–]
Community mobilization +structural interventions [n = 2]	STI cases [–]
Community self-efficacy [n = 1]	Self-negotiation [+]
Training on communication + relationship skills [n = 2]	Forced sex [–]
Structural intervention [n = 1]	Sex work [–]

The factors influencing HIV service uptake for female sex workers were awareness about HIV risk and consequences, fear of exposure to family, friends, and peers about involvement with the sex trade, quality of care in the available services, confidentiality issues in accessing services, accessibility to services including long distance, charges of public services [11]. Formal education, family counselling, peer‑based behavioural rehabilitation, and state-specific intervention strategies could reduce HIV transmission among IDUs in northeastern states [32]. One study from Manipur and Nagaland found that a comprehensive, integrated women’s health centre is more likely to attract women drug and alcohol users than the current HIV prevention services or existing government health services [12]. Prevention programmes targeted to Female Sex Workers (FSW) in Karnataka have successfully reduced risky behaviours, STIs, and HIV [52]. Utilization of HIV counselling services increased awareness about HIV and decreased STI incidence significantly among FSWs [67]. Evaluation of the Aastha program aimed to develop collective self-efficacy to prevent HIV/ STI, indicating that improved collective self-efficacy among the program participants helped increase negotiation power among FSWs. They could turn away clients unwilling to use condoms [68]. Evaluation of the Alliance intervention in Andhra Pradesh found that Sex workers with a high degree compared with a low degree of overall collectivization were significantly more likely to report high self-efficacy to use government health facilities (75.0% vs 57.3%, adjusted OR 2.5, 95% CI 2.0 to 3.1) and to use government health centres for STI treatment in past one year (78.1% vs 63.2%, adjusted OR 2.1, 95% CI 1.6 to 2.8), irrespective of a project partnership with government centres [69].

Three studies discussed the evaluation framework [70], monitoring tool [71] and impact [72] of a flagship HIV prevention program, the AVAHAN model of community mobilization for core population groups in four states–Maharashtra, Tamil Nadu, Karnataka, and Andhra Pradesh. There is a positive impact of the program suggesting that HIV prevalence declined significantly from 17.0% to 14.2% (P <0.001) from 2005–2006 to 2008–2009, pre- to post-intervention [72].

### Quality assessment of published studies

Most of the included quantitative studies have reported high selection bias (Refer to S3 Table). More than half of the quantitative studies reported risk of any other bias, including social desirability bias, non-response bias, recall bias or selection bias. The study samples did not represent the target population in approximately one-fourth of the included studies. However, the sample size was adequate in more than 90% of studies. Almost none of the studies reported the reliability and validity of the data tools. More than two-thirds of the studies reported confidence intervals, standard errors, or p-values in reporting their results, indicating robust reporting of results—however, none of the quantitative studies satisfied all the ROB assessment criteria. We could not conduct a meta-analysis because of study design and outcomes heterogeneity.

Half of the included qualitative studies either did not describe the methodology, did not report the underlying theory, or both (Refer to S4 Table). Furthermore, several studies did not straightforwardly elaborate on data collection settings. Approximately two-thirds of the studies did not mention any non-participation or dropout rates. Very few studies reported interview guides or field notes. Only one-third of the studies described coding trees or reported on the number of data coders. None of the included studies conducted participant checking, implying inadequate information on the qualitative data analysis. It is to be noted that five articles were excluded from the ROB assessment as they were poster presentations, abstracts, conceptual frameworks, or review articles, thereby limiting our opportunity to assess them constrained by the standard assessment tools.

## Discussion and conclusion

We identified 96 peer-reviewed studies that addressed transmission network or geographical concertation of sexual risk behaviour and STI/HIV infection (RQ1), awareness, prevalence, and incidence of STI / HIV (RQ2), determinants of sexual risk behaviour, STI/ HIV (RQ3), and health-seeking behaviour and intervention mechanisms to address STI/ HIV (RQ4) among AGYW community in the Indian context. Of these, 3, 23, 22 and 27 studies focused exclusively on RQ1, RQ2, RQ3 and RQ4, respectively. A few studies addressed more than one research objective. Studies used quantitative, qualitative, and mixed methods to analyze the research questions.

A high-quality systematic review would be ideal for addressing the research questions in this context. However, we attempted to conduct a rigorous rapid review given the time and resource constraints. The other limitations of the study are the following. We considered the documents published only in English within a specific time (2000–2023). We also did not conduct a meta-analysis due to the heterogeneity of study findings.

Limited evidence was available on the transmission network or high concentration areas for HIV/STI infection among the AGYW community in India. Studies pointed out low awareness about sexual risk behaviour/ STI/ HIV, and the prevalence and incidence of STI/ HIV are also low. However, the FSWs, IDUs, and human traffic victims suffer more. Prevalence of STI/RTI ranged from 11–17% in Andhra Pradesh, Maharashtra, Rajasthan and Jharkhand to 21–22% in Bihar and Tamil Nadu among the study participants [60]. The overall prevalence of HIV infection among the general population in India was reportedly 0.24% (95% CI: 0.21%–0.28%) [73].

Key determinants influencing sexual risk behaviours and the vulnerability of AGYW to STIs and HIV are primarily rooted in socioeconomic contexts, relationship dynamics, proximal factors, substance use, and awareness levels about STIs/HIV [30, 39, 44, 56, 73–79]. Socioeconomic factors such as age, educational attainment, marital status, economic status, and overall living conditions significantly dictate the vulnerability of AGYW to these health risks [4, 10]. Notably, younger individuals with lower educational backgrounds and those living in impoverished conditions are at heightened risk due to restricted access to relevant information and healthcare resources [2, 10]. The marital status of AGYW further influences their risk, with unmarried or younger married women often being more vulnerable due to reduced sexual autonomy and limited ability to negotiate within sexual relationships [9].

Furthermore, the power dynamics within partner relationships, including the age and educational background of the partner, play a pivotal role in determining the vulnerability of STI/HIV [10]. Power imbalances and the lack of negotiating capacity for safer sexual practices, such as consistent condom use, amplify these risks [10]. Proximal risk factors like early marriage, premature sexual initiation, and trafficking also constitute significant risk determinants. Such behaviours are often observed in environments where comprehensive sexual health education is absent, leading to risk-prone sexual behaviours [3, 10].

The consumption of substances and alcohol has been linked to risky sexual behaviours, including unprotected sex and having multiple sexual partners, thereby escalating the risk of STI/HIV transmission [12]. Awareness and knowledge about STIs and HIV are also crucial in shaping sexual behaviours. A lack of awareness, particularly prevalent in low-income settings, increases vulnerability [7, 33]. Misconceptions and misinformation about HIV/STIs compound this vulnerability [33].

These interrelated determinants highlight the complexity of vulnerability among AGYW, underscoring the need for multi-pronged intervention strategies. It is essential to implement educational initiatives, foster community engagement, and provide healthcare services that are specifically tailored to the needs of AGYW to mitigate these risks and reduce the prevalence of STIs and HIV [2, 9, 10].

Fifteen studies have delved into this area, revealing that factors such as age, caste, education level, economic status, and exposure to marital violence significantly influence these behaviours. Notably, AGYW tends to rely more on traditional sources for health information and advice, such as mothers, other female family members, friends, peers, and neighbours, rather than formal healthcare sources like public and private health facilities, STI clinics, or drop-in centres [7, 9]. This preference for informal sources may be attributed to accessibility, perceived confidentiality, and cultural norms surrounding sexual health [33].

In addition to understanding health-seeking behaviours, twenty-five studies have evaluated the impact of single or multiple-component interventions on various outcomes related to HIV knowledge, prevalence, and sexual risk-taking behaviours among AGYW. These interventions range from mass media campaigns and community mobilization efforts to structural interventions and life skills training [2, 10]. These strategies have been found effective in enhancing HIV-related knowledge, increasing condom usage, and improving self-efficacy in sexual health decisions among AGYW. For instance, by leveraging platforms like television, radio, and social media, mass media campaigns have been instrumental in disseminating crucial information about HIV prevention and safe sexual practices [12].

Community mobilization initiatives, which involve grassroots-level engagement and empowerment, have been shown to foster a supportive environment for AGYW to discuss and address sexual health issues. These initiatives also contribute to reducing the stigma associated with HIV and promoting healthy sexual behaviours [68, 71]. Structural interventions, which address broader socioeconomic factors influencing health behaviours, have been impactful in creating safer environments for AGYW, thereby reducing their vulnerability to STIs and HIV [10, 80].

Life skills training, which focuses on imparting knowledge about sexual health, negotiation skills, and decision-making, could be effective in equipping AGYW with the tools to navigate complex social and relational dynamics that influence their sexual health. These interventions highlight the importance of comprehensive and holistic policies in tackling these public health challenges by addressing the multifaceted nature of AGYW’s vulnerability to HIV and STIs.

### Conclusion

Despite significant government and NGO initiatives spanning the last two decades aimed at preventing HIV and STIs, both global and national data underscore the high vulnerability to these diseases among AGYW worldwide, including in India. Reports from surveillance and research in India have pinpointed adolescents, particularly girls, as an emerging high-risk and susceptible demographic. It is, therefore, imperative to document the prevalence, underlying factors, and healthcare-seeking patterns of AGYW concerning HIV/STIs at this juncture. This comprehensive rapid review offers valuable insights into the prevalence and diverse socioeconomic and geographic determinants of HIV/STI vulnerability among AGYW in India. This information can serve as a valuable resource for shaping and implementing effective policies geared towards preventing HIV/STIs in this population while also encouraging safe sexual practices and promoting positive health-seeking behaviours among AGYW in India.

In conclusion, our study warrants the implementation of appropriate gender (and age)-based culturally sensitive approaches to cope with the increasing vulnerability of young adults, especially girls, towards STIs/HIV in India. Future research may focus on identifying the transmission network or geographical concentration of the vulnerability of sexual risk behaviour/ STI/HIV infection among the AGYW in India, which would help effectively implement interventions. It would be beneficial to have detailed mixed-method studies to understand the sexual risk behaviour patterns of this vulnerable age group.

## Supporting information

S1 Checklist PRISMA 2020 for abstracts checklist. (DOC)

S2 Checklist PRISMA 2020 checklist. (DOCX)

S1 Appendix (PDF)

S1 File (DOCX)

S1 Table Final search strings. (DOCX)

S2 Table Summary of findings from all included studies in the rapid review. (XLSX)

S3 Table Quality assesment tool for quantitative studies. (XLSX)

S4 Table Quality assesment tool for qualitative studies. (XLSX)
