# Supplementary material for: Vulnerability to Sexually Transmitted Infections (STI) / Human Immunodeficiency Virus (HIV) among adolescent girls and young women in India: A rapid review
Source: PLoS One. 2024 Feb 14;19(2):e0298038. doi: 10.1371/journal.pone.0298038 (PMC10866498; doi:10.1371/journal.pone.0298038)
Supplement: S1 Checklist — (DOC) [file pone.0298038.s001.doc]

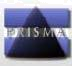
**PRISMA 2020 for Abstracts Checklist**

**For the paper**

**Vulnerability to Sexually Transmitted Infections (STI) / Human Immunodeficiency Virus (HIV) Among Adolescent Girls and Young Women in India: A rapid review**

|  |  |  |  |  |  |  |  |  |  |  |  |
| --- | --- | --- | --- | --- | --- | --- | --- | --- | --- | --- | --- |
|  | **Section and Topic** |  |  | **Item** |  |  | **Checklist item** |  |  | **Reported** |  |
|  |  |  |  | **#** |  |  |  |  |  | **(Yes/No)** |  |
|  |  |  |  |  |  |  |  |  |  |  |  |
|  |  | |  |  |  |  |  |  |  |  |  |
|  | **TITLE** | |  |  |  |  |  |  |  |  |  |
|  | Title | | 1 | |  |  | Identify the report as a rapid review. | |  | Y |  |
|  |  | |  |  |  |  |  |  |  |  |  |
|  |  | |  |  |  |  |  |  |  |  |  |
|  | **BACKGROUND** | |  |  |  |  |  |  |  |  |  |
|  | Objectives | | 2 | |  |  | Provide an explicit statement of the main objective(s) or question(s) the review addresses. | |  | Y |  |
|  |  | |  |  |  |  |  |  |  |  |  |
|  |  | |  |  |  |  |  |  |  |  |  |
|  | **METHODS** | |  |  |  |  |  |  |  |  |  |
|  | Eligibility criteria | | 3 | |  |  | Specify the inclusion and exclusion criteria for the review. | |  | Y |  |
|  |  | |  | |  |  |  | |  |  |  |
|  | Information sources | | 4 | |  |  | Specify the information sources (e.g. databases, registers) used to identify studies and the date when each | |  |  |  |
|  |  |  |  |  |  |  | was last searched. | |  | Y |  |
|  |  | |  | |  |  |  | |  |  |  |
|  | Risk of bias | | 5 | |  |  | Specify the methods used to assess risk of bias in the included studies. | |  | Y |  |
|  |  | |  | |  |  |  | |  |  |  |
|  | Synthesis of results | | 6 | |  |  | Specify the methods used to present and synthesise results. | |  | Y |  |
|  |  | |  |  |  |  |  |  |  |  |  |
|  |  | |  |  |  |  |  |  |  |  |  |
|  | **RESULTS** | |  |  |  |  |  |  |  |  |  |
|  | Included studies | | 7 | |  |  | Give the total number of included studies and participants and summarise relevant characteristics of studies. | |  | Y |  |
|  |  | |  | |  |  |  | |  |  |  |
|  | Synthesis of results | | 8 | |  |  | Present results for main outcomes, preferably indicating the number of included studies and participants for | |  |  |  |
|  |  |  |  |  |  |  | each. If meta-analysis was done, report the summary estimate and confidence/credible interval. If comparing | |  | Y(meta analysis not done) |  |
|  |  |  |  |  |  |  | groups, indicate the direction of the effect (i.e. which group is favoured). | |  |  |  |
|  |  | |  |  |  |  |  |  |  |  |  |
|  |  | |  |  |  |  |  |  |  |  |  |
|  | **DISCUSSION** | |  |  |  |  |  |  |  |  |  |
|  | Limitations of evidence | | 9 | |  |  | Provide a brief summary of the limitations of the evidence included in the review (e.g. study risk of bias, | |  |  |  |
|  |  |  |  |  |  |  | inconsistency and imprecision). | |  | Y |  |
|  |  | |  | |  |  |  | |  |  |  |
|  | Interpretation | | 10 | |  |  | Provide a general interpretation of the results and important implications. | |  | Y |  |
|  |  | |  |  |  |  |  |  |  |  |  |
|  |  | |  |  |  |  |  |  |  |  |  |
|  | **OTHER** | |  |  |  |  |  |  |  |  |  |
|  | Funding | | 11 | |  |  | Specify the primary source of funding for the review. | |  | Y |  |
|  |  | |  | |  |  |  | |  |  |  |
|  | Registration | | 12 | |  |  | Provide the register name and registration number. | |  | Y |  |
|  |  |  |  |  |  |  |  |  |  |  |  |
|  |  |  |  |  |  |  |  |  |  |  |  |

*From:* Page MJ, McKenzie JE, Bossuyt PM, Boutron I, Hoffmann TC, Mulrow CD, et al. The PRISMA 2020 statement: an updated guideline for reporting systematic

reviews. BMJ 2021;372:n71. doi: 10.1136/bmj.n71
