# Supplementary material for: Vulnerability to Sexually Transmitted Infections (STI) / Human Immunodeficiency Virus (HIV) among adolescent girls and young women in India: A rapid review
Source: PLoS One. 2024 Feb 14;19(2):e0298038. doi: 10.1371/journal.pone.0298038 (PMC10866498; doi:10.1371/journal.pone.0298038)
Supplement: S1 Appendix — (PDF) [file pone.0298038.s003.pdf]

Systematic review

Please select one of the options below to edit your record. Either option will create a new version of the record - the existing version will remain unchanged.

A list of fields that can be edited in an update can be found here

1. * Review title.

Give the title of the review in English

Vulnerability to Sexually Transmitted Infections (STI) / Human Immunodeficiency Virus (HIV) among adolescent girls and young women in India: A rapid review

2. Original language title.

For reviews in languages other than English, give the title in the original language. This will be displayed with the English language title.

Vulnerability to Sexually Transmitted Infections (STI) / Human Immunodeficiency Virus (HIV) among adolescent girls and young women in India: A rapid review

3. * Anticipated or actual start date.

Give the date the systematic review started or is expected to start.

15/03/2023

4. * Anticipated completion date.

Give the date by which the review is expected to be completed.

31/07/2023

5. * Stage of review at time of this submission.

This field uses answers to initial screening questions. It cannot be edited until after registration.

Tick the boxes to show which review tasks have been started and which have been completed.

Update this field each time any amendments are made to a published record.

The review has not yet started: Yes

Review stage	Started	Completed
Preliminary searches	No	No
Piloting of the study selection process	No	No
Formal screening of search results against eligibility criteria	No	No
Data extraction	No	No
Risk of bias (quality) assessment	No	No
Data analysis	No	No

Provide any other relevant information about the stage of the review here.

This will be a rapid review of literature
This will be a rapid review of literature

6. * Named contact.

The named contact is the guarantor for the accuracy of the information in the register record. This may be any member of the review team.

Sohini Paul

Email salutation (e.g. "Dr Smith" or "Joanne") for correspondence:
Dr Paul

7. * Named contact email.

Give the electronic email address of the named contact.

sohini.paul@gmail.com

8. Named contact address

PLEASE NOTE this information will be published in the PROSPERO record so please do not enter private information, i.e. personal home address

Give the full institutional/organisational postal address for the named contact.

Flat 503, Tower 1, Avanti Apartments, IIT Delhi Campus, Hauz Khas, New Delhi, 110016

9. Named contact phone number.

Give the telephone number for the named contact, including international dialling code.

9811756066

10. * Organisational affiliation of the review.

Full title of the organisational affiliations for this review and website address if available. This field may be completed as 'None' if the review is not affiliated to any organisation.

Population Council

Organisation web address:
https://popcouncilconsulting.com/

11. * Review team members and their organisational affiliations. [1 change]

Give the personal details and the organisational affiliations of each member of the review team. Affiliation refers to groups or organisations to which review team members belong.
NOTE: email and country now MUST be entered for each person, unless you are amending a published record.

Dr Sohini Paul. Population Council
Mr Anupam Sharma. Indian Institute of Technology, Gandhinagar, India
Ms Radhika Dayal. Population Council
Ms Sudeshna Maitra. Population Council
Ms Kuhika Seth. International AIDS Vaccine Initiative
Ms Monal Nagrath. International AIDS Vaccine Initiative
Dr Sowmya Ramesh. Population Council
Dr Niranjan Saggurti. Population Council
Ms Mahika Mehta. PopulationCouncil Consulting Pvt Ltd

12. * Funding sources/sponsors.

Details of the individuals, organizations, groups, companies or other legal entities who have funded or sponsored the review.

International AIDS Vaccine Initiative

Grant number(s)
State the funder, grant or award number and the date of award
A10622, Date of award: 1st August 2022

13. * Conflicts of interest.

List actual or perceived conflicts of interest (financial or academic).

None

14. Collaborators.

Give the name and affiliation of any individuals or organisations who are working on the review but who are not listed as review team members. **NOTE: email and country must be completed for each person, unless you are amending a published record.**

15. * Review question.

State the review question(s) clearly and precisely. It may be appropriate to break very broad questions down into a series of related more specific questions. Questions may be framed or refined using PI(E)COS or similar where relevant.

1. What is the spatial distribution/concentration of HIV/ STI infection in India?
2. What is the prevalence and incidence of sexually transmitted infections / HIV among women with a special focus on the AGYW population?
3. What are the common determinants of STI, HIV infection and sexual risk behaviours among women with a special focus on the AGYW population?
4. What are the health-seeking behaviour and interventions (and their effectiveness) to prevent/communicate about STIs / HIV and sexual risk behaviours among AGYW in India? In other words, what has worked and what hasn’t in engaging AGYW in sexual and reproductive health programmes/ interventions? How to create a safe space and better engagement of AGYW on STI and HIV?

16. * Searches.

State the sources that will be searched (e.g. Medline). Give the search dates, and any restrictions (e.g. language or publication date). Do NOT enter the full search strategy (it may be provided as a link or attachment below.)

Search engines like JSTOR, PubMed, Google Scholar, ScienceDirect and Population Council Knowledge Commons will be used to search for articles published between January 1, 2000, and September 1, 2022, using the search terms like Adolescent Girls and Young Women, HIV, Sexual and Reproductive Health, Sexual and Reproductive Health Knowledge, Sexual and Reproductive Health Awareness, Sexual and Reproductive Health Risks, Behavioural Interventions and others. The searches will survey the titles, abstracts, keywords and full texts of the articles. If upon completion of the initial review, we find insufficient relevant publications, we will additionally consider searching in POPLINE and others. Also, we will scrutinize the reference lists of the reviewed articles.

17. URL to search strategy.

Upload a file with your search strategy, or an example of a search strategy for a specific database, (including the keywords) in pdf or word format. In doing so you are consenting to the file being made publicly accessible.

Or provide a URL or link to the strategy. Do NOT provide links to your search **results**.

Do not make this file publicly available until the review is complete

18. * Condition or domain being studied.

Give a short description of the disease, condition or healthcare domain being studied in your systematic review.

The review will focus on understanding the prevalence and incidence of HIV/AIDS and other STIS (such as syphilis, HPV, herpes, Gonorrhoea, chlamydia, etc.) among the adolescent girls and young women from India in the age group of 15-24 years. It will thus focus on the infections/diseases caused by unsafe sexual contact with their partners. It will also include the prevalence of awareness and knowledge of STIs/HIV along with their health-seeking behaviour (related to STIs and HIV) among the study group. Information on sexual and reproductive health that do not cover sexual contacts (such as urinary tract infection) will be excluded from our rapid review.

19. * Participants/population.

Specify the participants or populations being studied in the review. The preferred format includes details of both inclusion and exclusion criteria.

The participants will include adolescent girls and young women in the age group of 15-24 in India

20. * Intervention(s), exposure(s).

Give full and clear descriptions or definitions of the interventions or the exposures to be reviewed. The preferred format includes details of both inclusion and exclusion criteria.

This study will focus on understanding the following related to HIV/STIs among adolescent girls and young women (15-24 years) in India.

1. Prevalence of STIs/HIV: The proportion of AGYW (compared to men or women of other age groups) who have been diagnosed with STI/HIV in a given time period.
2. Incidence of HIV/STIs: HIV/STI incidence is expressed as the estimated number of persons newly infected with HIV/STI during a specified time period (e.g., a year), or as a rate calculated by dividing the estimated number of persons newly infected with HIV/STI during a specified time period by the number of persons at risk for HIV/STI infection.
3. (Un)safe sexual practices (or sexual behaviors): The prevalence and types of sexual practices that the study population adopts with their partners.
4. Knowledge and awareness of HIV/STIs: We will review articles that focus on the awareness and knowledge of STIs (different types, transmission mechanisms, safe sex practices, dos and don’ts, and so on) among the study population.
5. Health-seeking behaviors: This study will also include the prevalence and types of health-seeking behaviors (from service care providers, frontline workers, NGOs, relatives/family/friends, etc.) among the study population in regard to STIs/HIV infection.

21. * Comparator(s)/control.

Where relevant, give details of the alternatives against which the intervention/exposure will be compared (e.g. another intervention or a non-exposed control group). The preferred format includes details of both inclusion and exclusion criteria.

In general, the study will include articles that focus only on sexual and reproductive health (related to STIs/HIV) among adolescent girls and young women of the age group 15-24 years from India. However, if studies include samples from other genders (and other age groups), then comparative statements of prevalence and incidence of the outcomes will be included in our study.

22. * Types of study to be included.

Give details of the study designs (e.g. RCT) that are eligible for inclusion in the review. The preferred format includes both inclusion and exclusion criteria. If there are no restrictions on the types of study, this should be stated.

Studies that used Randomized Control Trials (RCTs), and quasi-experimental design (fixed-effects, regression discontinuity, instrumental variables analysis, and difference-in-difference analysis) will be included in our study. Further, studies that used both longitudinal data sets and cross-sectional datasets will be included but should be stated in the title or abstract. Qualitative studies that use different qualitative inquiry methods such as case studies, interviews, focused group discussions, and ethnography, will be included in our study. Literature review articles of empirical research will also be a part of our rapid review. Theoretical articles without empirical evidence will be excluded from our study.

23. Context.

Give summary details of the setting or other relevant characteristics, which help define the inclusion or exclusion criteria.

The rapid review will be limited to empirical studies from India that have focused on STIs/HIV among adolescent girls and young women (15-24 years) from India. This will include studies that have samples of the AGYW population. We will focus on articles that have a study sample of our target population from any of the diverse settings of India (rural/urban/peri-urban; diverse socioeconomic groups (caste, income, religion, education, migrants/non-migrants, and so on)). We will exclude articles that are from contexts other than India and which do not involve participants of our target population (adolescent girls and young women of 15-24 years).

24. * Main outcome(s).

Give the pre-specified main (most important) outcomes of the review, including details of how the outcome is defined and measured and when these measurement are made, if these are part of the review inclusion criteria.

1. Prevalence of STIs/HIV: The proportion of AGYW (compared to men or women of other age groups) who have been diagnosed with STI/HIV in a given time period.
2. Incidence of HIV/STIs: HIV/STI incidence is expressed as the estimated number of persons newly infected with HIV/STI during a specified time period (e.g., a year), or as a rate calculated by dividing the estimated number of persons newly infected with HIV/STI during a specified time period by the number of persons at risk for HIV/STI infection.
3. (Un)safe sexual practices (or sexual behaviors): The prevalence and types of sexual practices that the study population adopts with their partners.
4. Knowledge and awareness of HIV/STIs: We will review articles that focus on the awareness and knowledge of STIs (different types, transmission mechanisms, safe sex practices, dos and don’ts, and so on) among the study population.
5. Health-seeking behaviors: This study will also include the prevalence and types of health-seeking behaviors (from service care providers, frontline workers, NGOs, relatives/family/friends, etc.) among the study population in regard to STIs/HIV infection.

Measures of effect

25. * Additional outcome(s).

List the pre-specified additional outcomes of the review, with a similar level of detail to that required for main outcomes. Where there are no additional outcomes please state ‘None’ or ‘Not applicable’ as appropriate to the review

None

Measures of effect

26. * Data extraction (selection and coding).

Describe how studies will be selected for inclusion. State what data will be extracted or obtained. State how this will be done and recorded.

Reviewers will first independently screen study titles for relevancy. Within the spreadsheet, they will mark the “title & abstract relevancy” column in the master spreadsheet I, R, or CD, denoting the following: R = Article title is relevant; include in the abstract review. I =Article is irrelevant; exclude from abstract review. CD=There is not enough information to determine relevance; reviewers “cannot decide” from the title/abstract, whether the study falls within the inclusion criteria.

The first level of title relevancy review will be conducted by four reviewers. Any discrepancies between the categorization by the four reviewers will be noted. This will be followed by a second-level judicial review by a separate reviewer who will conduct quality checks of the studies with discrepancies and studies deemed ineligible. The studies deemed to have ‘relevant’ titles will next be reviewed for abstract relevancy using the same aforementioned procedures. Upon completion of the eligibility assessments, data from the spreadsheet will be used to complete the PRISMA Flow Diagram.

Studies deemed relevant by both title and abstract will be divided equally among the four reviewers. Data will be abstracted individually by each reviewer. If questions arise, the reviewer will consult the other reviewers and they will come to a consensus. All data will be abstracted directly into the master spreadsheet. The extraction sheet will include title, author(s), date of publication, type of publication (e.g., summary, synthesis, single study), number and type of included studies (if a summary or synthesis), settings and population studied, interventions implemented, outcomes measured and results, and if relevant to the question, whether results differed among subgroups, such as by gender, socioeconomic status, ethnicity, etc.

Our data extraction will be blinded (researchers will be unaware of author/journal details).

27. * Risk of bias (quality) assessment.

State which characteristics of the studies will be assessed and/or any formal risk of bias/quality assessment tools that will be used.

It is not applicable in this rapid review of literature

28. * Strategy for data synthesis.

Describe the methods you plan to use to synthesise data. This **must not be generic text** but should be **specific to your review** and describe how the proposed approach will be applied to your data.
If meta-analysis is planned, describe the models to be used, methods to explore statistical heterogeneity, and software package to be used.

We will conduct a narrative synthesis of the findings from the included studies (the articles that were found relevant to our research questions), structured around the type of intervention and its content, target population characteristics, and the type of outcome/exposures. We intend to provide analytical summaries of the intervention/exposure effects for each study. The summary notes developed from the qualitative studies included in our review will be presented along with relevant quotes (verbatim) described in the articles to substantiate the arguments. This form of review will rely primarily on the use of words and text to summarise and explain the findings of the synthesis. The synthesis will be structured around telling the story of the findings from the included studies.

29. * Analysis of subgroups or subsets.

State any planned investigation of ‘subgroups’. Be clear and specific about which type of study or participant will be included in each group or covariate investigated. State the planned analytic approach.

Wherever the relevant data are available, subgroup analyses will be done for individuals. We also plan to do a subgroup analysis by age (adolescent versus adults, by income groups, by caste groups, by religious groups, by education categories, urban/rural disparities, migrant/non-migrant status, etc.).

Since this is a qualitative synthesis, subgroup analyses may be undertaken but it is not feasible to specify all the relevant sub-groups in advance.

30. * Type and method of review.

Select the type of review, review method and health area from the lists below.

Type of review

Cost effectiveness	No
Diagnostic	No
Epidemiologic	No
Individual patient data (IPD) meta-analysis	No
Intervention	No
Living systematic review	No
Meta-analysis	No
Methodology	No
Narrative synthesis	Yes
Network meta-analysis	No
Pre-clinical	No
Prevention	No
Prognostic	No
Prospective meta-analysis (PMA)	No
Review of reviews	No
Service delivery	No

Synthesis of qualitative studies	No
Systematic review	Yes
Other	Yes

It will be a rapid review of the literature

Health area of the review

Alcohol/substance misuse/abuse	No
Blood and immune system	No
Cancer	No
Cardiovascular	No
Care of the elderly	No
Child health	No
Complementary therapies	No
COVID-19	No
Crime and justice	No
Dental	No
Digestive system	No
Ear, nose and throat	No
Education	No
Endocrine and metabolic disorders	No
Eye disorders	No
General interest	No

Genetics	No
Health inequalities/health equity	Yes
Infections and infestations	No
International development	No
Mental health and behavioural conditions	No
Musculoskeletal	No
Neurological	No
Nursing	No
Obstetrics and gynaecology	No
Oral health	No
Palliative care	No
Perioperative care	No
Physiotherapy	No
Pregnancy and childbirth	No
Public health (including social determinants of health)	Yes
Rehabilitation	No
Respiratory disorders	No
Service delivery	No
Skin disorders	No
Social care	No

Surgery	No
Tropical Medicine	No
Urological	No
Wounds, injuries and accidents	No
Violence and abuse	No

31. Language.

Select each language individually to add it to the list below, use the bin icon to remove any added in error.

English

There is an English language summary.

32. * Country.

Select the country in which the review is being carried out. For multi-national collaborations select all the countries involved.

India

33. Other registration details.

Name any other organisation where the systematic review title or protocol is registered (e.g. Campbell, or The Joanna Briggs Institute) together with any unique identification number assigned by them.
If extracted data will be stored and made available through a repository such as the Systematic Review Data Repository (SRDR), details and a link should be included here.
If none, leave blank.

34. Reference and/or URL for published protocol.

If the protocol for this review is published provide details (authors, title and journal details, preferably in Vancouver format)

No I do not make this file publicly available until the review is complete

35. Dissemination plans.

Do you intend to publish the review on completion?

Yes

We plan to publish the rapid review article in a peer-reviewed journal

36. Keywords.

Give words or phrases that best describe the review. Separate keywords with a semicolon or new line. Keywords help PROSPERO users find your review (keywords do not appear in the public record but are included in searches). Be as specific and precise as possible. Avoid acronyms and abbreviations unless these are in wide use.

37. Details of any existing review of the same topic by the same authors.

If you are registering an update of an existing review give details of the earlier versions and include a full bibliographic reference, if available.

38. * Current review status.

Update review status when the review is completed and when it is published.
New registrations must be ongoing so this field is not editable for initial submission.

Review_Ongoing

39. Any additional information.

Provide any other information relevant to the registration of this review.

This will be a rapid review of the literature

40. Details of final report/publication(s) or preprints if available.

Leave empty until publication details are available OR you have a link to a preprint (NOTE: this field is not editable for initial submission).
List authors, title and journal details preferably in Vancouver format.
