# Supplementary material for: Vulnerability to Sexually Transmitted Infections (STI) / Human Immunodeficiency Virus (HIV) among adolescent girls and young women in India: A rapid review
Source: PLoS One. 2024 Feb 14;19(2):e0298038. doi: 10.1371/journal.pone.0298038 (PMC10866498; doi:10.1371/journal.pone.0298038)
Supplement: S1 File — (DOCX) [file pone.0298038.s004.docx]

**Vulnerability to Sexually Transmitted Infections (STI) / Human Immunodeficiency Virus (HIV) Among Adolescent Girls and Young Women in India: A rapid review protocol**

**Sohini Paul^1*#a^, Anupam Sharma^2^, Radhika Dayal^1^, Mahika Mehta^1^, Sudeshna Maitra^1^, Kuhika Seth^3^, Monal Nagrath^3^, Sowmya Ramesh^4^, Niranjan Saggurti^5^**

**^1^Population Council Consulting Pvt Ltd, Delhi, India**

**^2^Indian Institute of Technology, Gandhinagar, Gujarat, India**

**^3^International AIDS Vaccine Initiative, Delhi, India**

**^4^Population Council Institute, Delhi, India**

**^5^Population Council, Delhi, India**

**^#a^Current Address: Zone 5A, India Habitat Center, Lodi Road, New Delhi – 110003, India**

***Corresponding author**

**E-mail: spaul@pcconsulting.co.in**

**Abstract**

Objective

Though HIV incidence has declined over the past decades in India, little is known about the vulnerability of Adolescent girls and young women (AGYW) to HIV and STI infection because of limited data and evidence availability. It is crucial to understand to promote effective disease control mechanisms in the backdrop of a lifestyle transition in an emerging market economy like India. We will conduct a rapid review of the literature to understand the sexual risk behaviour, spatial patterns of vulnerability and prevalence of STI/HIV infection, and health-seeking behaviour of AGYW in India.

Methods

We will conduct the review using appropriate inclusion and exclusion criteria and search terms. We will include quantitative, qualitative and mixed-method studies from India and search different databases (JSTOR, PubMed, Google Scholar, Science Direct and Population Council Knowledge Commons) published between January 2000 and March 2023. We will include evaluation studies using randomized control trials and quasi-experimental or descriptive designs published in English. The study participants should be adolescent girls and young women aged 15-24. The rapid review will exclude the studies focusing only on men or women outside the above age range. Independent reviewers will check data relevancy and will extract data using all relevant parameters. The rapid review will ensure the assessment of data quality by an independent reviewer. We will report the descriptive statistics and use the narrative synthesis method for data analysis.

Discussion

The rapid review will summarise evidence on the sexual risk behaviour of adolescent girls and young women in India and their vulnerability to STI/HIV infection. It will help to address the evidence gap in this critical area of research.

**Rapid Review Registration:**

**PROSPERO** CRD42023403713

**Background**

The risk of HIV transmission has been a serious health concern in India over the past decades. India has approximately 2.3 [1.8-3.0] million people living with HIV/AIDS (1); most of these are individuals over the age of 15 years (1). HIV in India reflects a differential burden across the states—the highest adult prevalence of HIV, which is above the national average of 0.22% [0.17-0.29%] in 2020, is noted among three states in the North-East region (1). Further, Maharashtra accounted for the highest number of people living with HIV, with 3.90 lakh people living with HIV in the state as of 2020 (1).

Unsafe sexual practices (1,4,5) resulting from gender discrimination, gender-based violence, and socioeconomic deprivation drive the HIV epidemic among women of all age groups across the globe (2,3).

Though there is evidence that a high proportion of adult women suffer from HIV infection, there is a scarcity of relevant data and evidence on the vulnerability of adolescent girls and young women (AGYW) to HIV risks in the Indian context. However, the potential reasons for vulnerability, including early marriage, unsafe sex, and sexual violence, is likely relatively high among the AGYW population. According to the fifth round of the National Family Health Survey (NFHS) report, almost 23% of women were reportedly married below the legal age. About 23.7% of girls aged 15-19 have had a live birth (6). Further, the median number of days of the last sexual encounter reported by girls (15-19 years) was 7.7 days (6). Additionally, only about 33.7% of women in the age group 20-24 years (who had two or more partners in the last 12 months) had reported using a condom during sexual encounters (6), and about 7.7% of ever-married girls aged 18-24 years have reported experiencing sexual violence (6).

Very few studies on the vulnerability of the AGYW population to HIV risks are available depicting the Indian scenario. A retrospective study correlated sexual risk behaviour with HIV transmission among adolescent girls (7). Adolescents aged 14-16 years are more likely to be HIV-reactive than those aged between (17-19 years) as pointed out in a small-scale case study from India (8). A pilot study from western India found that HIV-related health services and screening should be tailored to adolescents and young adults' needs (9). There is also a deficient level of awareness and Knowledge about HIV among this population (10,11). Interestingly, reporting sexually transmitted infections (STI) is higher among girls than boys (12).

To assess what is already known about the risky sexual behaviour among AGYW, the vulnerability to STI/ HIV infection, and prevention and cure of it in the Indian context, we aim to conduct a rapid review of the literature for the past two decades (2000-2023).

The objective of the rapid review is to summarize the recent information available on spatial patterns of vulnerability towards STI/HIV infection, sexual risk behaviours, incidence and prevalence of STI and HIV among the AGYW population, health-seeking behaviours, and existing and ongoing interventions to prevent STIs and HIV among AGYW in India. To the best of our knowledge, no literature review is available in this context. This review will help us to identify the gap in related literature and expand the scope of future research.

**Methods**

**Protocol and Registration**

We will conduct the rapid review following the guidance from the Cochrane Rapid Reviews Methods Group, March 2020 (13). The protocol was registered on the International Prospective Register of Systematic Reviews (PROSPERO). We will use an adapted version of the preferred reporting items for systematic reviews and meta-analysis protocols (PRISMA-P) for reporting this protocol since no standardized reporting formula is available for rapid review protocols.

**Inclusion/ Exclusion Criteria**

We will select peer-reviewed published articles, reports and grey literature following the inclusion criteria elaborated in Table 1.

**Search Strategy**

We developed the primary search strategy and selected search terms following relevant rapid and systematic reviews and publications on HIV infections among AGYWs (14, 15,16,17,18,19,20,21). Initially, two investigators (SP and AS) conceptualized the study, two investigators (AS and RD) developed the search strategy, and four of the investigators (SM, MM, MN and KS) refined it further. The group decided to focus on electronic databases, especially JSTOR, PubMed, Google Scholar, Science Direct and Population Council Knowledge Commons, for the published articles and grey literature. The initial search strings used MeSH terms to include publications on the sexual and reproductive health of the AGYW community, risky sexual behaviour and prevalence and incidence of HIV/STI following the prescribed eligibility criteria. Five reviewers (RD, AS, MM, SM and KS) reviewed the initial search strings to check whether they could be expanded further, and the entire study team created a final list of search strings. These search strings will be used to search articles from the relevant websites mentioned above. The research team will manually review the included publications reference list and have relevant articles.

**Table 1: Inclusion and Exclusion criteria**

| **Criterion** | **Include** | **Exclude** |
| --- | --- | --- |
|  |  |  |
| Language | Published in English | Not in English |
|  |  |  |
| Population | Adolescent girls and young women | Men |
|  | 15-24 age group | Girls below the age of 15 |
|  |  | Women more than age 25 |
|  |  |  |
| Spatial distribution | India - national-level studies and state-specific studies | Other than India |
|  |  |  |
|  |  |  |
| Knowledge and incidence of STI/ RTI/HIV | Knowledge of STI/RTI/HIV | Awareness/prevalence/incidence of any disease other than STI/RTI/HIV |
|  | Prevalence of STI/RTI/HIV |  |
|  | Incidence of STI/RTI/HIV |  |
|  | Sources of information for STI/RTI/HIV |  |
|  |  |  |
| Determinants of STI/ HIV | Focusing on determinants of STI/HIV/ sexual risk behaviour at the individual level, community or institution level, societal level | The direction of association between determinants and outcomes not clear or inconclusive |
|  |  |  |
| Health seeking behaviour | Formal service providers | Does not consider any of the providers |
|  | Traditional healthcare providers |  |
|  | Informal sources |  |
|  | Prevention programs |  |
|  | Participatory training programs |  |
|  |  |  |
| Interventions | Evaluation of single or multiple component interventions compared against appropriate counterfactual | Insufficient details on intervention |
|  | Outcomes will include the following - | Evaluation not adequately designed. |
|  | HIV knowledge and prevalence |  |
|  | Risky sexual behaviour |  |
|  | Forced sex |  |
|  | Self-efficacy/self-negotiation/ Positive gender attitude |  |
|  |  |  |
| Timing | Studies conducted between 1st January 2000 to 31st March 2023 | Studies conducted before 2000 or after March 2023 |
|  |  |  |
| Study design | Empirical studies from | Editorials |
|  | Randomised Control Trial | Commentaries |
|  | Quasi experimental design | Research Brief |
|  | Used cross-section or longitudinal data |  |
|  | Methods used - qualitative/ quantitative/ mixed |  |
|  | Systematic reviews |  |
|  | Published report |  |
|  | Published journal article |  |
|  | Grey literature |  |

**Data and analysis**

***Record selection***

Preliminary search documents will be stored in Mendeley (software for reference management), version 1.19.5. The Mendeley software will transfer all the screened studies into Microsoft Excel. Four reviewers will first independently screen study titles for relevancy (AS, RD, SM, MM); within the spreadsheet, they will mark the "title & abstract relevancy" column in the master spreadsheet R, I, or CD, denoting the following: *R* -Article title is relevant; include in the abstract review; *I* -Article is irrelevant; exclude from abstract review; *CD-*There is not enough information to determine relevance; reviewers "cannot decide" from the title/abstract, whether the study falls within the inclusion criteria. Any discrepancies between the categorization by the four reviewers will be noted. This will be followed by a second-level judicial review by the other two reviewers (KS and MN), who will conduct quality checks of the studies with discrepancies and deemed ineligible.

The studies deemed to have 'relevant' titles will be reviewed for abstract relevancy using the same procedures mentioned above by three reviewers (SP, SR and NS). Upon completing the eligibility assessments, data from the spreadsheet will be used to complete the PRISMA Flow Diagram (Appendix Figure 1).

Data Extraction

Studies deemed relevant by title and abstract will be divided equally among the six reviewers (RD, AS, MM, SM, KS, MN). Data will be extracted individually by each reviewer. If questions arise, the reviewer will consult the other reviewers, and they will come to a consensus.

All data for the parameters mentioned in Table 2 from each publication will be extracted directly into the master spreadsheet. Data from reviews and meta-analyses will be directly extracted for inclusion in our rapid review.

A data extraction tool will be developed in Microsoft Excel. It will be piloted on the initial ten studies and then finalized with all possible data validation checks to improve the consistency of data extraction quality. If there is confusion about the availability or suitability of data points, this will be referred to an independent reviewer (SP) for a decision. Two reviewers (SR and NS) will review the protocol independently.

Table 2: Fields to be extracted from selected publications

| **Category** | **Fields** |
| --- | --- |
| Publication identifier | Authors |
|  | Article Title |
|  | Year of Publication |
|  | Publication Type (peer-reviewed paper, report, research brief) |
|  | Publication date |
|  | Journal title with volume, issue, and pages |
|  |  |
| Study design and sites | Geography (India/ specific state) |
|  | Design (quantitative/ qualitative/ mixed methods) |
|  | Dates of data collection |
| Sampling strategy | Simple / Stratified random sampling / Purposive sampling / Convenience sampling |
|  |  |
| Population | Sample size |
|  | Age of the respondents |
|  | Risk group (key population - people who inject drugs, men who have sex with men, transgender people, sex workers; non-key population - general AGYW community |
|  |  |
| Transmission network | Sexual Risk Behaviour |
|  | Any other secondary outcome measured (effect size) |
|  | Awareness of HIV/STI/sexual risk behaviour (outcome measure/ effect size) |
|  | Prevalence/incidence of STI (outcome measure/ effect size) |
|  | Prevalence/incidence of HIV (outcome measure/ effect size) |
|  |  |
| Determinants | Description of sexual behaviour |
|  | Determinants of STI |
|  | Determinants of HIV |
|  | Determinants of sexual risk behaviour |
|  |  |
| Health seeking behaviour | Strategies to encourage health seeking |
|  | Sources of health seeking |
|  |  |
| Interventions | Type (Knowledge, behaviour change) |
|  | Intervention components |
|  | Exposure measure |
|  | Frequency of exposure |

##

## **Quality assessment**

The quality of the included studies will be assessed by a reviewer (SP) outside the core data extraction team. Upon completion of individual reviewer quality assessments, discrepancies in ratings among the reviewers will be noted. Discrepant ratings will be discussed among the reviewers and a seventh reviewer (SP) until a consensus is reached.

## **Data Synthesis and Analysis**

We anticipate the rapid review will find a wide range of studies across four broad research themes. We will categorize the findings across four research questions – transmission network of HIV/STI infection in India, awareness/prevalence/incidence of sexually risky behaviour/STI/HIV, determinants of sexually risky behaviour/HIV/STI and health-seeking behaviour and intervention to address the vulnerability towards sexual risk behaviour/HIV/STI. We will pool outcomes where possible and report mean values with confidence intervals where applicable. Given the broad scope of the review and anticipated heterogeneity of study outcomes, interventions and study designs, we may not be able to conduct a meta-analysis. Instead, we will describe the available data using descriptive statistics and narrative synthesis. We will report the range of prevalence and incidence of STI/HIV among the AGYW community and group the study findings on determinants of vulnerability across the individual level, institutional or community level and societal levels. We will also classify findings by similar types of interventions or outcomes. Assessment of the risk of bias is beyond the scope of this review.

We will document if there is a significant amendment of this protocol after publication and report those in the review results.

**Discussion**

We attempted to work on this rapid review based on a research call from International AIDs Vaccine Institute (IAVI) to understand the sexual risk behaviour and vulnerability towards HIV/STI among adolescent girls and young women in India as a background to their adaptability towards HIV and HPV vaccine. A complete application of the high-quality systematic review would be the ideal methodology. However, we intend to use the rapid review method because of time and resource constraints. As mentioned in the protocol, the other limitations of this study would be to consider the articles/ reports/ briefs published only in English and may not be able to conduct meta-analysis or risk of bias assessment.

The rapid reviews described in this protocol will summarize relevant literature on the transmission network of STI/HIV infections, prevalence, incidence and awareness of STI/HIV, determinants of sexual risk behaviour/STI/HIV, health-seeking behaviour and intervention strategies which worked or did not work to change the behavioural pattern among AGYW community in the Indian context. This will help us to identify the literature gap in this context and highlight the future scope of research in this crucial area.

**Acknowledgement**

We thank the experts (Key Informants) and study participants. The study would be impossible without their kind support.

**Author Contributions**

Conceptualization: SP, AS, RD; Methodology: SP, AS, RD, MM, SM, KS, MN; Writing – original draft: SP, RD, AS; Writing – Review and editing: KS, SR, NS

**Conflict of interest**

There is no conflict of interest

**Funding**

This project was funded by International AIDS Vaccine Institute (IAVI) under grant number AI0622.

**Data Availability**

The data will be made available upon request

**Ethics approval and consent to participate**

Not Applicable since it does not involve any human or animal experiment

**Consent for publication**

Not Applicable

***Appendix***

**Figure 1: PRISMA Flow Diagram**

Full-text articles excluded, with reasons
(n = )

Records excluded
(n = )

Additional records identified through other sources
(n = )

Records identified through database searching
(n = )

## Identification

Records after duplicates removed
(n = )

## Screening

Records screened
(n = )

Full-text articles assessed for eligibility
(n = )

## Eligibility

Studies included in qualitative synthesis
(n = )

Studies included in quantitative synthesis
(n = )

## Included

**Ackno**

project.

**References:**

1. AIDS Control Organization N. National AIDS Control Organisation, ICMR-National Institute Of Medical Statistics, Ministry of Health & Family Welfare, Government of India, Report 2021.
2. Greener R, Sarkar S. Risk and vulnerability: do socioeconomic factors influence the risk of acquiring HIV in Asia? AIDS [Internet]. 2010 Sep [cited 2022 Aug 16];24 Suppl 3(SUPPL. 3). Available from: https://pubmed.ncbi.nlm.nih.gov/20926925/
3. Shri N, Muhammad T. Association of intimate partner violence and other risk factors with HIV infection among married women in India: evidence from National Family Health Survey 2015–16. BMC Public Health [Internet]. 2021 Dec 1 [cited 2022 Aug 16];21(1):1–11. Available from: <https://bmcpublichealth.biomedcentral.com/articles/10.1186/s12889-021-12100-0>
4. UNAIDS. Key population groups, including gay men and other men who have sex with men, sex workers, transgender people and people who inject drugs[Internet]. [cited 2022 Aug 29]. Available from: https://www.unaids.org/en/topic/key-populations
5. Paranjape RS, Challacombe SJ. HIV/AIDS in India: an overview of the Indian epidemic. Oral Dis [Internet]. 2016 Apr 1 [cited 2022 Aug 16];22 Suppl 1:10–4. Available from: https://pubmed.ncbi.nlm.nih.gov/27109267/
6. International Institute for Population Sciences. National Family Health Survey - 5 2019-21. Ministry of Health and Family Welfare National. 2020;361.
7. Mehra B, Bhalla P, Rawat D. Indian adolescents and human immunodeficiency virus: A pilot study from Delhi. J Family Med Prim Care [Internet]. 2016 [cited 2022 Aug 16];5(1):187. Available from: /pmc/articles/PMC4943137/
8. Kurapati S, Vajpayee M, Raina M, Vishnubhatla S. Adolescents Living with HIV: An Indian Profile. AIDS Res Treat [Internet]. 2012 [cited 2022 Aug 16];2012. Available from: https://pubmed.ncbi.nlm.nih.gov/22778924/
9. Madan-Patel G, Mazumdar V. Clinical profile and disease progression of HIV in adolescents and young adults in Vadodara, India. Indian J Sex Transm Dis AIDS [Internet]. 2021 Jan 1 [cited 2022 Aug 16];42(1):24–30. Available from: https://pubmed.ncbi.nlm.nih.gov/34765934/
10. McManus A, Dhar L. Study of Knowledge, perception and attitude of adolescent girls towards STIs/HIV, safer sex and sex education: (a cross-sectional survey of urban adolescent school girls in South Delhi, India). BMC Women's Health [Internet]. 2008 Jul 23 [cited 2022 Aug 17];8. Available from: https://pubmed.ncbi.nlm.nih.gov/18647417/
11. Richard AK, Roland YK, Christian YK, Cécile KKA, Michel AJ, Lacina C, et al. Knowledge, Attitudes, and Practices of HIV-Positive Adolescents Related to HIV/AIDS Prevention in Abidjan (Côte d'Ivoire). Int J Pediatr [Internet]. 2020 [cited 2022 Aug 16];2020. Available from: /pmc/articles/PMC7785380/
12. Sinha A, Siddhanta A, Bartelsman M, Vaughan K, Rooijen V, Vries Hjc D, et al. P4.15 Knowledge &amp; awareness about sti-HIV and its prevalence among adolescents in India. Sex Transm Infect [Internet]. 2017 Jul 1 [cited 2022 Aug 29];93(Suppl 2): A197–A197. Available from: <https://sti.bmj.com/content/93/Suppl_2/A197>.
13. Garritty C, Gartlehner G, Nussbaumer-Streit B, King VJ, Hamel C, Kamel C, et al. Cochrane Rapid Reviews Methods Group offers evidence-informed guidance to conduct rapid reviews. J Clin Epidemiol. 2020. 130:13-22.
14. Brahme, R., Godbole, S., Sonawale, S., Kadu, C., Yadav, R., Dulhani, N., et al. Declining Trends in HIV Prevalence Among Women Attending Antenatal Care Clinics Obfuscate the Continued Vulnerability of Adolescent Girls in Maharashtra, India (2005-2017). J Acquir Immune Defic Syndr*.* 2019*;82*(1), e13-e17.
15. Budukh, A., Maheshwari, A., Palayekar, V., Bagal, S., Purwar, P., Deodhar, K., et al. Prevalence and nonsexual transmission of human papillomavirus (HPV) in the adolescent girls from rural area of Maharashtra state, India. Indian J Cancer*.* 2018; *55*(4), 336-339.
16. Devarayasamudram, S., De Gagne, J. C., Kurudi, N. P., & Kang, H. S. Effectiveness of a Structured Teaching Program on Knowledge and Attitudes Toward HIV among Young Women in India. J Community Health Nurs*.* 2018; *35*(2), 49-56.
17. Jejeebhoy, S. J., Santhya, K. G., & Acharya, R. Physical and sexual violence and symptoms of gynaecological morbidity among married young women in India. Glob Public Health*.* 2013; *8*(10), 1151-1167.
18. Nath, A. HIV/AIDS and Indian youth--a review of the literature (1980-2008). SAHARA J*.* 2009; *6*(1), 2-8.
19. Orchard, T. R. Girl, woman, lover, mother: towards a new understanding of child prostitution among young Devadasis in rural Karnataka, India. Soc Sci Med. 2007;  *64*(12), 2379-2390.
20. Pack, A. P., Sastry, J., Tolley, E. E., Kaaya, S., Headley, J., Kaale, A., et al. Stakeholder acceptability of adolescent participation in clinical trials for biomedical HIV prevention products: considerations from Tanzania and India. AIDS Care. 2019; *31*(7), 857-863.
21. Parchure, R., Darak, S., Jori, V., Hegde, A., Puri, A. K., Kulkarni, V., et al. Increasing sero-discordancy among young HIV infected pregnant women from India: a likely pointer of changing transmission dynamics. AIDS Care. 2019; 31(12), 1518-1526.
