# Supplementary material for: Vulnerability to Sexually Transmitted Infections (STI) / Human Immunodeficiency Virus (HIV) among adolescent girls and young women in India: A rapid review
Source: PLoS One. 2024 Feb 14;19(2):e0298038. doi: 10.1371/journal.pone.0298038 (PMC10866498; doi:10.1371/journal.pone.0298038)
Supplement: S1 Table — (DOCX) [file pone.0298038.s005.docx]

**Table: Final Search Strings**

| **Objectives** | **MeSH terms** |
| --- | --- |
| Spatial distribution/concentration of HIV/ STI^[[1]](#footnote-1)^ infection in India | Spatial OR  Geography |
| Prevalence and incidence of sexually transmitted infections / HIV among women with a special focus on the AGYW population | Prevalence OR  Epidemiology OR  Magnitude OR  Incidence **OR**  Burden OR  Sexually transmitted infections OR STI OR  Acquired immune deficiency syndrome OR HIV/AIDS OR  Sexual risk behaviours OR  Risky Behaviours OR  Unsafe sex/sexual practices OR  Risky sexual practices OR  Inconsistent condom use OR  People living with HIV/AIDS) OR PLWHA) AND antiretroviral therapy) OR ART) OR highly active antiretroviral therapy) OR HAART) |
| Determinants of STI, HIV infection and sexual risk behaviours among women with a special focus on the AGYW population? | Predictors OR  Determinants OR  Risk factors OR  Associated factors |
| Health-seeking behaviour and interventions (and their effectiveness) to prevent /communicate about STIs / HIV and sexual risk behaviours among AGYW in India | HIV prevention interventions OR  STI prevention interventions OR  Prevention OR  Preventive measures OR  Prevention and control OR  Interventions OR  Programmes OR |
| Study Population | Adolescent girls OR young women OR girls OR youth OR adolescents OR juvenile OR young people OR youth OR girl OR boy OR teen OR student OR young male OR young female OR female OR women OR adult women OR gender |
| Study Setting | India, search with adding names of states:  Andhra Pradesh; Arunachal Pradesh; Assam; Bihar; Chhattisgarh; Goa; Gujarat; Haryana; Himachal Pradesh; Jharkhand; Karnataka; Kerala; Madhya Pradesh; Maharashtra; Manipur; Meghalaya; Mizoram; Nagaland; Odisha; Punjab; Rajasthan; Sikkim; Tamil Nadu; Telangana; Tripura; Uttarakhand; Uttar Pradesh; West Bengal  **Union Territories:**  Andaman and Nicobar Island; Chandigarh; Daman and Diu; Jammu and Kashmir; Ladakh; Lakshadweep; Puducherry and Delhi |
| **Study Method** | Intervention OR program OR randomized control trial OR Quasi-experimental OR intervention* OR  experiment* OR trial* OR RCT quasi-experiment* OR observation* OR descriptive* OR longitudinal  evaluate* OR program* OR review* OR project* OR scheme* OR model* quantitative OR qualitative OR  mixed method |

1. STI infections include Gonorrhoea, Chlamydial Infections, Syphilis, Chancroid, Granuloma inguinale, Bacterial vaginosis, Candidiasis, Trichomoniasis, Herpes simplex virus infections, Human papilloma virus infection, Hepatitis B, Hepatitis folloiwng <http://naco.gov.in/sites/default/files/STI_Lab%20manual_09-01-2014.pdf> as accessed on 28th February 2023 [↑](#footnote-ref-1)
